# Implementation of a Cancer Navigation Intervention for Newly Diagnosed Survivors of Breast Cancer: Protocol for a Randomized Controlled Trial

**DOI:** 10.2196/85820

**Published:** 2026-04-20

**Authors:** Melba Sheila D'Souza, Ruby Gidda, Michelle Smith, Arati Swaminadhan, Amy Jean Strank, Ashwin Nairy

**Affiliations:** 1 Nursing and Population Health, School of Nursing, Thompson Rivers University, 805 TRU Way Kamloops, Kamloops, BC, V2C 0C8, Canada, 1 6047516672, 1 6047516672; 2 School of Nursing, University of British Columbia, Abbotsford, BC, Canada; 3 Clinical Capacity Optimization, BC Cancer Agency, Abbotsford, BC, Canada; 4 Faculty of Health and Social Development, UBC School of Health and Exercise Sciences, University of British Columbia, Kelowna, BC, Canada; 5 Learning Health Systems, Research Department, Interior Health, Kelowna, BC, Canada; 6 The University of British Columbia, UBC Experimental Medicine, University of British Columbia, Vancouver, BC, Canada; 7 Home Health, Interior Health, Kamloops, BC, Canada; 8 The University of British Columbia, Faculty of Science, University of British Columbia, Vancouver, BC, Canada

**Keywords:** oncology, cancer, cancer navigation, early survivorship, supportive care, digital health, rural communities, patient engagement, randomized controlled trial, mixed methods

## Abstract

**Background:**

Cancer inequities among vulnerable populations in rural areas remain a public health challenge in Canada. Rural populations are defined as vulnerable due to geographic isolation, limited access to specialized oncology care, and socioeconomic barriers such as transportation and financial toxicity. Professional navigation offers a potential solution to bridge these gaps, yet there is a lack of evidence on the barriers to and facilitators of its adoption in breast cancer survivorship.

**Objective:**

The objective of this study is to evaluate the effectiveness of a cancer navigation intervention using professional navigators compared to the standard of care (medical care) in improving the quality of life and functional outcomes of newly diagnosed survivors of breast cancer in interior British Columbia.

**Methods:**

A single-center, parallel-group, open-cohort randomized controlled trial is being conducted over 3 years. Ethics approval was obtained for the study. Participants who provide informed consent are randomized into 2 groups: the intervention group receives the cancer navigation intervention and the control group receives the standard of care (the usual medical care offered by health care practitioners). The baseline study time point spanned January to March 2025, the first follow-up spanned April to June 2025 at 3 months after enrollment, and the second follow-up spanned July to September 2025 at the end of 6 months after enrollment. The cancer navigation intervention comprises direct psychosocial and educational webinars, coordinated telephone support services, and community-based cancer care resources. Professional navigators are qualified registered nurses who facilitate information and connect participants with available supportive resources, services, and programs. The main outcomes are financial distress, quality of life, and satisfaction with navigation and interpersonal relationships. The Comprehensive Score for Financial Toxicity–Functional Assessment of Chronic Illness Therapy, Functional Assessment of Cancer Therapy–Breast, Breast Cancer Navigation Survey, Participant Satisfaction With Navigation Scale, Satisfaction With Interpersonal Relationships Survey, and Breast Cancer Navigation Interview are used in the study. Steps are being taken to ensure the trustworthiness of the qualitative data. With a 5% level of significance (2 tailed) and 90% power, the sample size was calculated as 108.

**Results:**

Data collection took place from January 2 to September 30, 2025. A total of 164 participants were recruited.

**Conclusions:**

This study aims to demonstrate effectiveness and satisfaction with professional navigation and knowledge translation for future implementation of a cancer navigation intervention in British Columbia.

## Introduction

### Background

Cancer is Canada’s leading cause of mortality, affecting not only individuals but also the health care system [1]. According to the Canadian Cancer Society, 2 in 5 Canadians are expected to be diagnosed with cancer in their lifetime; approximately 1 in 4 Canadians was expected to die of the disease in 2024 [2]. In 2022, a total of 2.3 million women worldwide were diagnosed with breast cancer, resulting in 670,000 deaths [3]. As of 2024, breast cancer is still the most commonly diagnosed cancer among Canadian women [2]. The Canadian Cancer Society estimates that 30,500 women will receive a breast cancer diagnosis in 2024, accounting for 25% of all new cancer cases in women. Approximately 5500 women are expected to die from the disease in 2024, which represented 13% of all cancer-related deaths among Canadian women in 2024 [2]. Early diagnoses, prompt referrals, and timely access to appropriate treatments are associated with better reported outcomes.

Breast cancer accounts for 28% of all cancer diagnoses for women and is the most common cancer in this population [4]. Ensuring equitable access to cancer care, whereby individuals receive care based on their needs, is crucial for timely treatment and the effective navigation of complex care pathways [5]. A cancer diagnosis poses challenges, and patient navigation programs assist individuals and their families in managing the complexities of survivorship. Cancer treatment involves complex services and diverse health care professionals in various units, which challenges equitable care and emphasizes the need to identify where disparities occur along the care continuum [5]. Socioeconomic factors, such as the lack of insurance and transportation as well as financial concerns, affect the access to and use of cancer care services [6]. While survival rates have improved, the transition from active treatment to survivorship care is often fragmented.

Patient navigation programs have been shown to address physical, emotional, and financial challenges through interventions such as psychosocial counseling, educational webinars, and coordinated access to community-based resources [7]. Access to and the use of health care services remains crucial for individuals following cancer treatment and throughout posttreatment care [8]. There are variations in access to cancer care after treatment and supportive care in rural areas [9]. Patient navigation programs play a crucial role in enhancing reported outcomes and addressing treatment inequities stemming from nonmedical factors [10]. Fewer interventions address psychosocial well-being and the navigation of health care services for adults diagnosed with breast cancer [11]. Hence, it is important to target reported outcomes, increase access to supportive care, and increase understanding of supportive care [10].

Approximately 17% of the population resides in British Columbia [12]. In interior regions, rural isolation, limited health care resources, and inadequate access to specialized care often lead to poorer reported outcomes [13]. Black individuals, Asian individuals, and those from other minority ethnic groups, as well as those from low-income and remote areas, likely face delayed cancer treatment [14]. Early findings from one study show that 24% of participants reported not receiving adequate emotional support during their breast cancer treatment journey and 50% felt adequately supported at diagnosis [15]. A total of 24% of survivors report insufficient emotional support.

Several studies have demonstrated positive associations between patient navigation and ameliorated distress, quality of life (QOL), and anxiety [16-19]. Patients aged 65 years and younger and those residing in rural areas reported significantly lower distress scores at discharge, supporting the value of incorporating oncology nurse navigators into care pathways [20]. People from socioeconomically disadvantaged groups in rural settings experience unique challenges in accessing care, which can impact reported outcomes [21]. The lack of continuity often leads to a limited awareness of care. Moreover, in another study, 24% of women reported insufficient emotional support during their breast cancer journey, whereas only 50% felt adequately supported at diagnosis [22]. In one study, 20% of women expressed a desire for more emotional support [6]. In another study, engagement in a patient navigation program led to improvements in distress and QOL over 18 months among participants, positively influencing patients’ well-being [23]. Survivors of cancer often face psychosocial issues that can harm QOL and may decrease adherence to follow-up care [24]. Health care providers should assess psychosocial and mental health distress and provide appropriate referrals to counseling or support groups. Several factors impact patients living with cancer, such as difficulty navigating services, a lack of follow-up, and inadequate care coordination [25]. Addressing the complex needs of survivors of cancer requires an integrated approach that encompasses supportive and survivorship care planning, psychosocial support, and effective communication among health care providers [26].

Patient navigators serve as liaisons within the health care system, performing core functions, such as providing patient education, offering emotional support, coordinating logistics, and facilitating access to resources [27]. Patient navigators assist patients in understanding their care plans while addressing practical challenges such as transportation, scheduling, and financial concerns [28]. A systematic review found that patient navigator interventions decreased delays in care among survivors of cancer [29]. Improving accessibility and health literacy requires tailored interventions and consistent follow-up, informed by an assessment of the needs of immigrant and refugee women [30]. Studies have demonstrated that, with proper implementation and evaluation, patient-reported outcomes, satisfaction, and self-efficacy in underserved communities can be improved [31,32]. Despite the continued lack of culturally and linguistically appropriate content for equity-deserving populations, the Functional Assessment of Cancer Therapy–Breast (FACT-B) remains a vital tool to measure QOL across four domains: physical, social, emotional, and functional well-being.

### Aim

The aim of this study is to evaluate the effectiveness of a cancer navigation intervention compared to the standard of care (SOC) in improving QOL and functional outcomes for newly diagnosed survivors of breast cancer in interior British Columbia.

## Methods

### Research Design

This study uses implementation science through an open-cohort randomized controlled trial (RCT) involving an intervention and a control group. Implementation science is defined as the promotion of the systematic uptake of research findings and other evidence-based practices into routine practice to improve the quality and effectiveness of health services [33,34]. An RCT is a study in which participants are randomly allocated to receive a new treatment (experimental group), a standard treatment (comparison group), or no treatment at all (placebo group) [35]. This RCT is a single-center, parallel-group study with stratified block randomization that follows the CONSORT (Consolidated Standards of Reporting Trials) guidelines (Checklist 1) and assesses a cancer navigation intervention provided by professional navigators.

### Participants and Setting

Study participants were recruited from Kamloops, Revelstoke, Merritt, 100 Mile House, Kelowna, Vernon, Penticton, and Okanagan, all situated in interior British Columbia.

### Eligibility Criteria

The inclusion criteria are adults older than 18 years who can read, understand, and write in English or their preferred primary language; are newly diagnosed with and being treated for breast cancer; and are willing to participate in the study.

The exclusion criteria are adults with advanced cancer, secondary cancer, or concurrent malignancies; neonates, infants, or children younger than 19 years; and self-reported severe, undiagnosed, or untreated neurological, psychiatric, and cognitive problems impairing understanding and provision of informed consent.

### Recruitment and Data Collection

Recruitment for the study took place with permission from local groups, institutions, and establishments. The recruitment call was distributed through printed posters, social media, community networks, and online advertisements. During the initial recruitment conversation, the research assistant described the study project, informing participants of what is expected, reading the study information sheet, and providing clarification. Participants were screened for eligibility and randomized into the study. Participants who met the criteria were enrolled via a web link or QR code.

**Figure 1.**
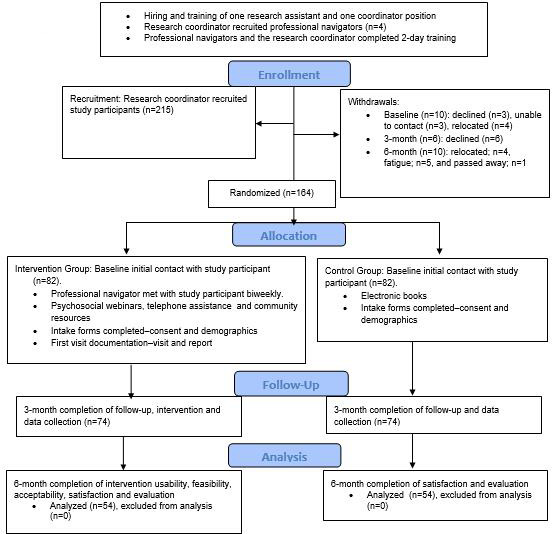
CONSORT (Consolidated Standards of Reporting Trials) flow diagram.

Participants who provided informed consent were randomized (1:1) into one of the two study groups: the intervention group received the cancer navigation intervention and the control group received the SOC (the usual medical care offered by health care practitioners; Figure 1). The computer-based randomization balanced random assignments to the two groups in blocks of 5 participants. The baseline study time point spanned January 2025 to March 2025, the 3-month follow-up spanned April 2025 to June 2025, and the 6-month follow-up spanned July 2025 to September 2025.

### Intervention and Control Groups

The cancer navigation intervention comprises direct psychosocial and educational webinars, coordinated telephone support services, and community-based cancer care resources offered by professional navigators (Table 1). Professional navigators are qualified registered nurses trained in professional navigation to provide information, connect patients with available supportive services, and help identify information for survivorship. Participants engage in navigator follow-ups, ensuring continuous access to professional guidance.

**Table 1.** Cancer Navigation Reported Outcomes research protocol.

Activity	Protocol	Expectation
1. Recruit eligible participants	Advertisement for participant recruitment: recruit participants by advertisement and poster announcements. When: weekly	Review eligibility criteria to yield eligibility determination. When: at the time of initial participant contact
2. Initial contact by the research team	Cover letter to participants and informed consent form and code of conduct: meet with participants via phone for study information, an introduction, and the baseline survey. When: within 1 week of recruitment or scheduling of the initial appointment	Speak to participants by phone for informed consent and the baseline survey: sociodemographic survey (5 minutes), COST-FACIT^a^ (5 minutes), and FACT-B^b^ (10 minutes) When: within 1 week of initial participant recruitment appointment
3. Communication with the navigator	Participant permission script: communicate with the navigator by email and phone at regular intervals regarding the participant’s enrollment and check-ins. When: within 1 week following randomization	Communicate with the navigator by email and phone at scheduled appointments When: within 1 week following randomization
4. Tracking participants over time	Coordinated telephone support service: meet with participants online based on the schedule. When: within 1 week of randomization	When: (1) at initial intake, (2) within 1 month of randomization, and (3) at the end of each month until completion Speak to a participant by phone at least 6 times during navigation, a total of 12 sessions.
5. Number of contact attempts to reach the participant	Attempt to reach participants by the preferred contact method, usually by phone at least 3 times, including one in the “evening” hours, followed by 1 follow-up letter from the research team. When: once daily (1 attempt/day for 3 different days)	Attempt to reach participant by preferred contact method, usually by phone at least 3 times on 3 different days When: once daily (one attempt/day for 3 different days)
6. Missed appointment follow-up	Any missed appointments should result in a navigator phone call and completion of the navigation activity. If the participant has a history of missed appointments, the navigator should provide a reminder call the day before every scheduled appointment. When: within 48 hours of missed appointment	After the first missed appointment, the navigator should call to determine the cause and complete an assessment if determined necessary. When: within 1 week of missed appointment
7. Participants lost to follow-up	Message participants, and reach out to the research team. When: after 3 contact attempts, being unable to reach the participant, and not showing up for scheduled appointments	Call the participant. When: after 3 contact attempts, being unable to reach the participant, and not showing up for the scheduled appointments
8. Completing the navigation	Complete direct psychosocial and educational webinars: interactive teaching-learning and interactive webinars When: end of 3 months and at the end of 6 months	When: within 1 month of randomization and at the end of 3 months until completion Interaction with the participant online at least two times during navigation, a total of 4 sessions. Interactive webinars with the participant online at least 2 times, a total of 4 sessions.
9. Follow-up	Schedule of enrollment, trial, and metrics: the navigator should ask participants at each follow-up encounter if their identified needs have been met and documented.	Follow-up by phone When: 1 week after assessment

a COST-FACIT: Comprehensive Score for Financial Toxicity–Functional Assessment of Chronic Illness Therapy.

b FACT-B: Functional Assessment of Cancer Therapy–Breast.

Participants were randomized to either the cancer navigation intervention or the SOC (the control group). The cancer navigation intervention took place biweekly over 3 months and was facilitated by a professional navigator. The intervention follows tailored approaches to care based on full assessments of participants’ needs and preferences. The intervention includes a full assessment, information and emotional support, and care planning specific to breast cancer survivorship. Professional navigators interact with participants in person during the intervention and follow up with them to provide psychoeducational webinars, telephone helpline support, and cancer care resources (Table 1). The intervention comprises 15 minutes of telephone calls biweekly for 3 months and 6 months, 45 minutes of interactive teaching-learning, a 30-minute webinar, and a 50-minute participant interview, totaling 5 hours (Table 2).

**Table 2.** Schedule of enrollment, trial, and metrics (outcomes) of the intervention and control groups.

	Study period and time point			
	Enrollment (T1), n	Postallocation (T1 at baseline; 0 weeks)	Follow-up (T2 at the end of 3 months; 12 weeks)	Evaluation (T3 at the end of 6 months; 12 weeks)
Enrollment, eligibility screening, informed consent, and randomization allocation	164	^—a^	—	—
Cancer navigation intervention: intervention group	82	—	—	—
Telephone calls	82	6 sessions	6 sessions	—
Interactive teaching-learning	82	2 sessions	2 sessions	—
Interactive webinar	82	2 sessions	2 sessions	—
Standard of care: control group	82	No sessions	No sessions	—
Interview and surveys				
Sociodemographic survey	164	5 minutes	5 minutes	5 minutes
Comprehensive Score for Financial Toxicity–Functional Assessment of Chronic Illness Therapy	164	10 minutes	—	10 minutes
The Functional Assessment of Cancer Therapy–Breast	164	5 minutes	—	5 minutes
Breast Cancer Navigation Survey	164	10 minutes,	—	10 minutes
Participant Satisfaction With Navigation Survey	164	10 minutes	—	10 minutes
Satisfaction With Interpersonal Relationships Survey	164	10 minutes	—	10 minutes
Breast Cancer Navigation Interview	82	—	—	45‐50 minutes

a Not applicable.

### Data Measurement Instruments and Outcomes

The main outcomes are financial distress; QOL, such as physical, social, emotional, and functional well-being; and satisfaction with navigation and interpersonal relationships, measured using the instruments outlined in this section.

A sociodemographic survey is used to collect the demographics of the participants.

The COST-FACIT was developed by de Souza et al [36] in conjunction with the University of Chicago and is a patient-reported outcome measure that describes the financial distress experienced by patients with cancer.

The FACT-B is a 37-item instrument designed to measure 5 domains of health-related QOL in patients with breast cancer: physical, social, emotional, and functional well-being and a breast cancer subscale. The FACT-B was developed with an emphasis on patients’ values and brevity [37].

The Breast Cancer Navigation Survey (BCNS) comprises 10 items on a 5-point rating scale from “very dissatisfied” to “very satisfied” and 3 items on a 3-point rating scale from “very dissatisfied” to “very satisfied” [38]. This survey takes 10 minutes to complete (Multimedia Appendix 1).

The Participant Satisfaction With Navigation Scale (PSNS) has 26 items on a 5-point rating scale, from “very satisfied” to “do not know” or “refuse to answer,” was developed by a multidisciplinary team of researchers and clinicians from the National Cancer Institute (NCI)–sponsored Patient Navigation Research Program, led by Jean-Pierre [39]. This survey takes 10 minutes to complete.

The Satisfaction With Interpersonal Relationships Survey comprises 10 items on a 5-point rating scale from “very dissatisfied” to “very satisfied” and 3 items on a 3-point rating scale from “very dissatisfied” to “very satisfied” [40]. This survey takes 10 minutes to complete.

The Breast Cancer Navigation Interview includes 10 questions on perspectives on diagnosis, treatment, and navigation (Multimedia Appendix 2).

Internal consistency reliability for the COST-FACIT for participants diagnosed with breast cancer using Cronbach α for the 2-factor structure was 0.76 to 0.82. A pretest with 30 female patients with breast cancer found a mean COST-FACIT score of 21.23 (SD 9.85). Construct validity for the COST-FACIT score correlated negatively with higher psychological distress and positively with better QOL and lower out-of-pocket costs.

Internal consistency reliability for the Functional Assessment of Cancer Therapy–Breast (FACT-B) total score was Cronbach α >0.90, with subscale Cronbach α values for the physical, social, emotional, and functional domains being between 0.71 and 0.83 for participants diagnosed with breast cancer. The tool exhibited concurrent validity with the EORTC QLQ-C30 and high sensitivity to clinical changes, identifying significant shifts in performance status and disease stage via a 6- to 7-point score variance.

Internal consistency reliability for the BCNS using the Cronbach α was 0.70 to 0.78 when pretesting with participants diagnosed with breast cancer. Face and content validity were established through an expert panel consisting of 5 members: one oncologist, one physician, one nurse, one patient, and one caregiver.

Internal consistency reliability for the PSNS using the Cronbach α was 0.81 when pretesting among participants diagnosed with breast cancer. Predictive validity showed that higher satisfaction scores correlated with lower levels of decisional conflict and higher adherence to follow-up treatments. The mean satisfaction score was 3.7 out of 5.0.

Internal consistency reliability for the Satisfaction With Interpersonal Relationships Survey ranged from α=0.75 to α=0.84 among participants with breast cancer. The tool exhibited discriminant validity by differentiating between technical competence and interpersonal attributes. Satisfaction regarding trust was associated with emotional well-being subscales.

### Trustworthiness

To ensure the trustworthiness of the qualitative findings, the researchers followed the criteria of credibility, dependability, confirmability, and transferability. Credibility and confirmability were preserved through reflexivity and bracketing; researchers used reflexive journaling to critically examine and set aside preconceived notions, ensuring the findings emerged directly from the lived experiences of participants. To establish dependability, an audit trail was carefully maintained to document and track all analytical decisions. These strategies enhanced the transferability of the work, providing a rigorous framework for the study.

### Sample Size and Power

The study is sufficiently powered to detect a clinically meaningful difference while accounting for the longitudinal design. For effect size, the power assumption calculation was based on a minimum clinically significant difference of 7 points on the FACT-B [41]. An estimated SD of 16.93 was used, derived from existing literature on adult survivors of cancer. An attrition rate of 25% was estimated based on the potential for dropout during the 6-month follow-up schedule. While the study is a single-center RCT, the randomization is balanced using blocks of 5 to mitigate potential clustering effects and ensure equal distribution across the 2 arms. With a 5% level of significance (2 tailed) and 90% power, the required sample size was calculated as 108 participants. The total recruitment target is set to 150 participants (75 in the intervention group and 75 in the control group).

### Ethical Considerations

The study received an approval certificate from the BC Cancer Agency, Interior Health Authority, and Thompson Rivers University Research Ethics Board (H22-03105/27-04-2023/27-04-2024). Research team members completed the Tri-Council Policy Statement: Ethical Conduct for Research Involving Humans, a course on research ethics. Participants will receive a gift voucher for their time and efforts at baseline and after 3 months.

### Quality, Safety, and Monitoring

The research assistants developed a master sheet, which includes the study location and site; participant codes (study ID); follow-up tracking; the participant’s name and contact information (email, phone number, and address); the randomization arm; date and time and modules completed; and whether the intervention is in progress or incomplete. Research assistants reminded participants via email and phone to review intervention module information. Discussions among the research team addressed any adverse effects that could result from study participation, the validity and integrity of the data, the enrollment rate relative to expectations, participant characteristics, retention, adherence to the protocol (potential or real deviations), and data completeness. We have established a data and safety monitoring plan to ensure participants’ safety. These activities are reviewed by screening results and other available data at weekly research meetings among the principal investigator, the research team, and other relevant research team members; in a quarterly review of data safety and enrollment by the research team, including the principal investigator, research assistants, and relevant research team members; and in an annual review by the ethics board.

### Data Analysis

All quantitative data will be analyzed using the intention-to-treat principle. Participants who engaged with the study from October 2025 to December 2025 will be included in the analysis.

The FACT-B is the primary measure used to evaluate the effectiveness of professional navigation on health-related QOL. Changes in FACT-B total scores and the scores on its 5 subscales (physical, social, emotional, and functional well-being and the breast cancer subscale) from baseline (T1) to 3 months (T2) and 6 months (T3) will be compared between the cancer navigation intervention and SOC control groups. A repeated-measure ANOVA will be used to determine the interaction between time and the treatment group. A change of 6 to 7 points on the FACT-B is considered clinically significant for this population.

Financial toxicity is an indicator of socioeconomic distress. Financial burden will be quantified using the COST-FACIT instrument. Descriptive statistics will be generated. To determine the impact of financial toxicity on health-related QOL, ANOVAs will be conducted to assess the associations between COST-FACIT scores and health-related QOL outcomes.

The BCNS analyzes participant satisfaction across 13 items to evaluate the user-friendliness and clarity of the program objectives.

The PSNS uses 26 items to measure overall satisfaction with the navigation process. Higher scores will be correlated with secondary outcomes such as treatment adherence using predictive validity testing.

The Satisfaction With Interpersonal Relationships Survey evaluates soft skills and trust in the professional navigator. Discriminant validity will be assessed by distinguishing between technical competence and interpersonal satisfaction scores.

Qualitative data from the Breast Cancer Navigation Interview will be analyzed using the thematic analysis approach by Braun and Clarke [42] to identify barriers to and facilitators of the adoption of professional navigation. These qualitative findings will be synthesized with the quantitative results to explain the how and why behind the intervention and effectiveness data. Two independent researchers will read the transcripts multiple times to achieve immersion. Participant quotations will be analyzed in terms of the content; what was being discussed; context; language use, such as metaphors, symbols, repetitions, and pauses; and initial interpretative comments by the participants [43]. Researchers will look at associations among emerging themes and group them according to conceptual similarities; track them; log them; and then extract major themes, subthemes, and relevant excerpts from the interview transcripts. Codes will be generated to capture emerging concepts and grouped into clusters based on conceptual similarities to categorize themes.

## Results

### Study Timeline

Data collection took place from January 2 to September 30, 2025. Funding was secured in June 2022; the study has progressed through data validation and cleaning; publication of the results is anticipated in December 2026. A total of 164 participants were recruited at baseline and randomized into either the intervention group (n=82) or the control group (n=82), as shown in Figure 1. At the end of the 6-month follow-up, 108 participants had completed the study (54 in the intervention group and 54 in the control group), resulting in a response rate of 65.85%.

### Sociodemographic Characteristics

In the intervention group (n=54), the largest age group was 40-49 years (17/54, 31.48%), whereas the control group (n=54) was slightly older, with the largest age group being 50-59 years (24/54, 44.44%). Educational attainment was comparable across both groups, with individuals who had completed college, associate, or technical levels comprising 33.33% (18/54) of the intervention group and 37.04% (20/54) in the control group.

Differences were observed in employment and residency. The control group reported a higher employment rate (25/54, 46.3%) than the intervention group (16/54, 29.63%). A higher percentage of the intervention group resided in rural areas (44/54, 81.48%) compared to the control group (30/54, 55.56%).

### Functional Assessment of Cancer Therapy–Breast

At the 6-month follow-up, participants in the intervention group demonstrated higher mean scores across all FACT-B domains than the control group. The intervention group reported higher mean scores (mean 25.6, SD 5.2) on the breast cancer subscale than the control group (mean 24.5, SD 7.1; *P*<.01), indicating improved QOL outcomes.

### Comprehensive Score for Financial Toxicity–Functional Assessment of Chronic Illness Therapy

Age group (*P*=.05), education level (*P*<.05), employment status (*P*=.01), and place of residence (*P*<.03) were significantly associated with COST-FACIT scores.

## Discussion

The study seeks to identify how navigation can enhance supportive and survivorship care and anticipates improving QOL. This study illustrates findings that emphasize the necessity of addressing the transition to survivorship through dedicated psychosocial support. The trial is recruiting participants from rural communities, yet it remains a single-center study, which may limit the generalizability of the findings to other provinces. The effectiveness of the study components may be hindered by varying levels of health literacy. The inclusion criteria allow participants who speak English or their preferred primary language, but the availability of culturally and linguistically tailored services remains a challenge for equity-deserving groups. A longitudinal design over 6 months introduces the possibility of attrition, which could impact the analysis even with the use of the intention-to-treat principle.

This RCT plans to establish an intervention framework for evaluating the effectiveness of a cancer navigation intervention in improving QOL and functional outcomes for newly diagnosed survivors of breast cancer. The study aims to demonstrate how professional navigation can bridge the gap between medical care and the psychosocial needs of rural and vulnerable populations. This study seeks to provide a contextual, evidence-informed model for professional navigation to enhance patient satisfaction. The study will demonstrate that professional navigators enhance trust and communication, reinforcing the value of person-centered care. Data will be disseminated through community engagement forums that will aim to influence health policy and evidence synthesis regarding breast cancer survivorship.

## Supplementary material

10.2196/85820 Multimedia Appendix 1 Breast Cancer Navigation Survey and Participant Satisfaction With Navigation Scale.

10.2196/85820 Multimedia Appendix 2 Breast Cancer Navigation Interview.

10.2196/85820 Checklist 1 CONSORT checklist.

## Abbreviations

BCNS: Breast Cancer Navigation Survey
CONSORT: Consolidated Standards of Reporting Trials
COST-FACIT: Comprehensive Score for Financial Toxicity–Functional Assessment of Chronic Illness Therapy
FACT-B: Functional Assessment of Cancer Therapy–Breast
PSNS: Participant Satisfaction With Navigation Scale
QOL: Quality of life
RCT: Randomized controlled trial
SOC: Standard of care
