# Supplementary material for: Implementation of a Cancer Navigation Intervention for Newly Diagnosed Survivors of Breast Cancer: Protocol for a Randomized Controlled Trial
Source: JMIR Res Protoc. 2026 Apr 20;15:e85820. doi: 10.2196/85820 (PMC13094795; doi:10.2196/85820)
Supplement: Multimedia Appendix 1 [file resprot-v15-e85820-s001.pdf]

Questionnaires and Scales

BREAST CANCER NAVIGATION

Title of the project

Enhancing cancer navigation for newly diagnosed, treated and post-treatment of people living with breast cancer in interior region of British Columbia.

Short title: Cancer navigation experiences of people living with breast cancer

Project abbreviation: Cancer Navigation and Reported Outcomes (CNRO)

Principal Investigator: Dr. Melba D'Souza, Email: mdsouza@tru.ca, Mobile. 604751 6672

Study instructions: This survey will take 30 minutes. The breast cancer navigation has three parts: Breast Cancer Navigation Survey (BCNS), Participant Satisfaction With Navigation Scale (PSNS) and Satisfaction With The Interpersonal Relationship (SIPR).

Part 1. BREAST CANCER NAVIGATION SURVEY (BCNS)

Name of Participant ____

Participant ID (write a 5-digit, use the first two letters of the month and three numbers 0-9, for example, JU-217): _______-

We value your input into the cancer navigation intervention to better meet your needs. Please circle your response; please add comments for a choice of 3 items or below.

**Participant Survey**	**Very Dissatisfied**	Dissatisfied	**Neutral**	Satisfied	**Very Satisfied**
1. I received adequate information pertaining to education	1	2	3	4	5
2. My calls were returned in a timely manner	1	2	3	4	5
3. I felt the navigator knew about navigation	1	2	3	4	5
4. The navigator provided me with helpful information during my care	1	2	3	4	5
5. Support services referrals met my needs	1	2	3	4	5
6. I was satisfied with instructions and had responsive answers to questions	1	2	3	4	5
7. The navigator was thorough and kept me informed	1	2	3	4	5
8. I valued working with the navigator	1	2	3	4	5
9. I found the participant breast cancer education materials helpful	1	2	3	4	5
10. I would recommend this breast cancer education to others	1	2	3	4	5

11. Please give your overall rating of the Navigation. Would you say you were…
    Very Dissatisfied Neutral Very Satisfied
12. How would you rate your overall experience with the navigation? Would you say you were…
    Very Dissatisfied Neutral Very Satisfied
13. Did having navigation and being part of this navigation keep you from seeking care elsewhere?

Suggestions or comments:

____

____

Part 2. PARTICIPANT SATISFACTION WITH NAVIGATION SCALE (PSNS)

1. Participant ID (write a 5-digit, use the first two letters of the month and three numbers 0-9, for example, JU-217): _______-

The following questions ask about problems you may have faced in getting needed health care since you were told you had a breast cancer. For each problem, indicate whether you were very satisfied (very happy), a little satisfied (happy for the most part), or not satisfied (not happy) with the help you received from your navigation. Some things may not apply to you. If I ask about something that was never a problem for you, just say so (not a problem) and we will skip it.

	**Very Satisfied**	**A Little Satisfied**	**Not Satisfied**	**Not a Problem N/A**	**Don't Know/ Refuse to answer**
14. making appointments with the navigator	3	2	1	0	98
15. understanding what you were being told to do about your care	3	2	1	0	98
16. getting results of tests you had	3	2	1	0	98
17. dealing with financial concerns related to getting the care you need	3	2	1	0	98
18. getting transportation to the doctor's office	3	2	1	0	98
19. feeling less overwhelmed by your health issues	3	2	1	0	98
20. giving you emotional support	3	2	1	0	98
21. encouraging you to talk to the doctor about your concerns	3	2	1	0	98
22. dealing with fears related to your health issues	3	2	1	0	98
23. getting the health information you needed	3	2	1	0	98
24. making you more involved in decisions about your health care	3	2	1	0	98
25. dealing with personal problems related to your health	3	2	1	0	98
26. dealing with work or employer issues related to health care	3	2	1	0	98
27. understanding the medical tests you got	3	2	1	0	98
28. understanding your health issues	3	2	1	0	98
29. knowing who to call when you had a question	3	2	1	0	98
30. learning about services in the community that are available to you	3	2	1	0	98
31. dealing with housing and landlord issues if appliable	3	2	1	0	98
32. dealing with the paperwork	3	2	1	0	98
33. understanding letters, reports, and health education materials	3	2	1	0	98
34. getting the services in the community that you are eligible for	3	2	1	0	98
35. getting childcare or eldercare so that you could go to your doctors' appointments	3	2	1	0	98
36. dealing with health insurance matters	3	2	1	0	98
37. including family members in the care you received	3	2	1	0	98
38. dealing with doctors, nurses, and other healthcare workers who do not speak your language	3	2	1	0	98
39. overcoming barriers related to a physical disability	3	2	1	0	98

Part 3. SATISFACTION WITH THE INTERPERSONAL RELATIONSHIP (SIPR)

The next nine statements are related to your satisfaction with the interpersonal relationship with your navigator(s). For these next statements, I will read the statement to you and I want you to tell me if you "strongly disagree," "disagree," "are undecided," "agree," or "strongly agree" with the statement I am going to read."

I feel my Navigator…	Strongly Disagree	Disagree	Undecided	Agree	Strongly Agree	Don't Know/ Refuse to answer
40. is easy to talk to	1	2	3	4	5	98
41. listens to my problems	1	2	3	4	5	98
42. is dependable	1	2	3	4	5	98
43. is easy for me to reach	1	2	3	4	5	98
44. cares about me personally	1	2	3	4	5	98
45. is courteous and respectful to me	1	2	3	4	5	98
46. gives me enough time	1	2	3	4	5	98
47. figures out the important issues in my health care	1	2	3	4	5	98
48. makes me feel comfortable	1	2	3	4	5	98

Thank you for your participation.
