# Supplementary material for: Implementation of a Cancer Navigation Intervention for Newly Diagnosed Survivors of Breast Cancer: Protocol for a Randomized Controlled Trial
Source: JMIR Res Protoc. 2026 Apr 20;15:e85820. doi: 10.2196/85820 (PMC13094795; doi:10.2196/85820)
Supplement: Multimedia Appendix 2 [file resprot-v15-e85820-s002.pdf]

BREAST CANCER NAVIGATION

BREAST CANCER NAVIGATION INTERVIEW (BCNI)

27 December 2024, Version 3

Title of the project
Enhancing cancer navigation for newly diagnosed, treated and post-treatment of people living with breast cancer in interior region of British Columbia.
Short title: Cancer navigation experiences of people living with breast cancer
Project abbreviation: Cancer Navigation and Reported Outcomes (CNRO)
Principal Investigator: Dr. Melba D'Souza, School of Nursing, Thompson Rivers University, Email: mdsouza@tru.ca, Mobile. 604 751 6672
Study instructions: This is a culturally safe, comfortable and accessible space to share your voice. This interview will take 45-50 minutes and is recorded for transcription. The recording will be destroyed immediately upon completion of the study. You can choose to skip the questions, stop and/or end the interview at any time. You can access the community resources if needed. This interview will not influence the medical and health care you are receiving at any time.
Part A. Perspectives of diagnosis and treatment in our own words or opinion

1. How did you think you were informed about the diagnosis of breast cancer?
2. What do you think about the diagnosis of breast cancer and the treatment plan?
3. What/who do you feel influences your decision to be treated for breast cancer?
4. How would you think you received information about the diagnosis of breast cancer?

Part B. Experience of navigation in your own words or opinion

1. What do you think the experience of attending a professional navigation session meant to you?
2. What do you think was beneficial to you for attending the professional navigation session?
3. What do you think are areas of improvement for the professional navigation session? In other words, what you do think can help to make it better?
4. What do you think are the main things that influenced your decision to attend a professional navigation session?
5. What do you think was helpful about the experience you had at the professional navigation session?
6. How do you think professional navigation sessions affected you in terms of what you learned or gained personally from participating?
